# Sepsis-Associated Encephalopathy

**Published:** 2012-01-18

**Authors:** Simona Cotena, Ornella Piazza

**Affiliations:** 1 Dipartimento di Scienze Chirurgiche, Anestesiologiche Rianimatorie e dell’Emergenza, Università degli Studi di Napoli “Federico II”.; 2 Dipartimento di Anestesia e Rianimazione, Università degli Studi di Salerno.

**Keywords:** Sepsis-associated encephalopathy, S100B, blood brain barrier, brain edema

## Abstract

Sepsis-associated encephalopathy (SAE) is defined as a diffuse or multifocal cerebral dysfunction induced by the systemic response to the infection without clinical or laboratory evidence of direct brain infection.

Its pathogenesis is multifactorial. SAE generally occurs early during severe sepsis and precedes multiple-organ failure.

The most common clinical feature of SAE is the consciousness alteration which ranges from mildly reduced awareness to unresponsiveness and coma.

Diagnosis of SAE is primarily clinical and depends on the exclusion of other possible causes of brain deterioration. Electroencephalography (EEG) is almost sensitive, but it is not specific for SAE. Computed Tomography (CT) head scan generally is negative in case of SAE, while Magnetic Resonance Imaging (MRI) can show brain abnormalities in case of SAE, but they are not specific for this condition. Somatosensitive Evoked Potentials (SEPs) are sensitive markers of developing cerebral dysfunction in sepsis. Cerebrospinal fluid (CBF) analysis is generally normal, a part an inconstant elevation of proteins concentration.

S100B and NSE have been proposed like biomarkers for diagnosis of SAE, but the existing data are controversial. SAE is reversible even if survivors of severe sepsis have often long lasting or irreversible cognitive and behavioral sequel; however the presence of SAE can have a negative influence on survival.

A specific therapy of SAE does not exist and the outcome depends on a prompt and appropriate treatment of sepsis as whole.

The brain is not, as once thought, a privileged organ protected by BBB, but it is strongly interconnected to the immune system and it is actively involved in the response to a noxious stimuli like an infection.

As an example, we may think to a condition in which fever is present, the most simple and studied model of the inflammatory response of the brain.

The term SAE is used to define a diffuse or multifocal cerebral dysfunction induced by the systemic response to the infection without clinical or laboratory evidence of direct brain infection (1).

We shortly review SAE epidemiology, the main patho-physiologic theories, its clinical presentation, diagnosis, prognosis and the management.

## Pathogenesis of SAE

SAE occurs in 9% to 71% of all critical ill patients suffering from sepsis (2). This wide range is due to the definition criteria extremely variable among the different Authors.

The pathogenesis of SAE is unclear; several mechanisms have been proposed like possible causes of brain damage during severe sepsis: the direct bacterial invasion, the effect of endotoxins and of the inflammatory mediators, the BBB dysfunction and the CBF impairment, the alteration of amino acids and of neurotransmitters, apoptosis, oxidative stress and excitotoxicity, but the most accredited hypothesis is that pathogenesis of SAE is multifactorial. The following paragraphs discuss these possible mechanisms.

### Direct bacterial invasion

Although in many cases of severe sepsis, a cerebral localization of micro-organisms had been described, the term SAE, instead of septic encephalopathy, stresses the concept that the brain impairment which often occurs in septic patients is independent from a direct cerebral infection. In fact, many cases of SAE without micro-abscesses have been observed and there is no correlation between SAE and a particular microorganism (3). So, it is unlikely that direct brain infection may play a causative role in SAE.

### Endotoxins

Bacterial toxins and, in particular, LPS (lypopolisaccharide), are more likely one of the causes of brain dysfunction during sepsis. According to Sharshar LPS in high concentrations could act directly on the brain in the circumventricular organs which are not protected by the BBB. Here LPS would bind its receptors such as the Toll Like Receptor (TLR), inducing the synthesis of inflammatory cytokines, prostaglandines and Nitric Oxide (NO) from microglia and astrocytes.

At low concentrations it would induce the peripheral secretion, from monocytes/macrophages, of inflammatory cytokines, among which IL-6 would directly act on the brain inducing the expression of mediators of the inflammation (4).

### Inflammatory mediators

When an infection occurs, peripheral monocytes/macrophages respond with the secretion of inflammatory cytokines; among these IL-1, TNF-α and IL-6 seem to be the most important ones in mediating the cerebral response to infection. While IL-1 and TNF-α have a local action inducing IL-6 production, this last one has endocrine properties and plays a key role in brain activation (3). In fact it is able to induce COX2 (cyclooxygenase 2) from glial cells and so the synthesis of prostaglandins and in particular of prostaglandin E2 which is responsible of the activation of hypothalamus pituitary adrenal axis (HPAA), of fever and of behavioral alterations (5). The activation of the complement cascade, particularly of the anaphylatoxin C5a, has been associated to brain dysfunction during sepsis, probably by inducing BBB break down (6).

### BBB dysfunction

Both LPS and cytokines are able to induce an endothelial activation named panendothelitis. In fact, they induce the expression of adhesion molecules on brain microvessel endothelial cells; they also induce the secretion of proinflammatory cytokines and of nitric oxide synthase (NOS). This endothelial activation results in increased permeability and disruption of BBB with consequent vasogenic brain edema. Furthermore, astrocytes endfeet surrounding cortical microvessels are frequently swollen and their membranes ruptured and detached from the microvessel walls. Astrocyte endfeet swelling is a direct consequence of the BBB breakdown; on the other hand, it may further impair BBB function in sepsis. Perimicrovessel edema impairs the movement of oxygen, nutrients and metabolites across the microvessel wall.

As direct consequence of BBB dysfunction, the differential flux of chemical is altered in sepsis; aromatic amino acids are more readily transported than branched chain amino acids from blood to brain; this results in an increased ratio of aromatic to branched-chain amino acids in plasma (see below).

Is not clear if the vasogenic edema found in MRI images of patients with SAE is the consequence of BBB disruption. In according to some Authors (7) some other mechanism might be involved in the formation of edema. Brain edema in SAE may be related to the loss of autoregulation rather than to BBB damage even if the initial vasogenic edema may evolve into cytotoxic edema (8).

### CBF and cerebrovascular autoregulation

CBF decrease and brain ischemia have been considered possible causes of brain impairment during severe sepsis, even if cerebral dysfunction occurs also in absence of hemodynamic alterations. An impairment of CBF could be the consequence of microvascular alterations, which occur in other organs, rather than an effect of systemic hypotension.

Several Authors measured CBF in septic patients, but the results are almost controversial; while some Authors found an effective decrease of CBF, other ones found it normal or increased (9–10).

Up to date, it is not clear if in patients with severe sepsis there is a loss of cerebral autoregulation and if it could may play a role in the pathogenesis of SAE (8).

In a recent study by Szatmári and colleagues an impaired cerebral vasomotor reactivity studied by acetazolamide test was found. This reduced reactivity was attributed by the Authors to a vasoconstriction at the level of resistance arterioles due to many factors: the disruption of the BBB which allows the passage of endogenous cathecolamines; the inhibition of endothelial NOS by cytokines; microthromboses and microinfarctions resulting from the alteration of the coagulation system (11).

### Amino acids and Neurotransmitters imbalance

In septic patients plasma an increased ratio of aromatic to branched-chain amino acids (AAA/BCAA) has been found. This is probably the consequence of the severe catabolic state which characterizes sepsis. In these conditions, in fact, there is an intense protheolysis with release in the plasma of both aromatic and branched-chain amino acids; these latter are used from the muscle for energy production.

Systemic inflammation also causes an increase of the cerebral release and of the unidirectional influx of phenylalanine. The consequent increased arterial levels of this aminoacid may affect cerebral function by reducing the availability of BCAAs to the brain. In particular the reduction of the influx of leucine can impair excitatory neurotransmission.

Furthermore, it has been demonstrated a net cerebral efflux of glutamine after LPS infusion, which probably reflects an its compensatory astrocytic release in order to reduce osmotic stress, or an increased cerebral proteolysis (12).

An imbalance of amino acid ratio has been shown even in the CSF and it is caused, like previously reported, by BBB dysfunction.

An increase of serum concentration of gamma-aminobutyric acid (GABA) has been proposed as alternative mechanism of brain dysfunction in sepsis, but the data existing in literature are controversial (1).

### Mitochondrial dysfunction

Mitochondrial dysfunction may be related to both the induction of neural cell apoptosis and an insufficient energy supply.

A decrease in mitochondrial ATP generation is caused by cytokines, reactive oxygen species (ROS) and NO. On the other hand, mitochondria can induce neuronal apoptosis by releasing cytocrome C (13).

### Calcium homeostasis

SAE is associated to an increase in intracellular calcium levels. This increase has been attributed to the ischemia, to the presence of excitatory aminoacids or to a direct effect of cytokines. The alteration of calcium homeostasis impairs learning memory and cognitive function (13).

In conclusion none of the described mechanism plays as an isolated causative mechanism of SAE but a multifactorial action should be verified. Moreover, the brain dysfunction during sepsis could be also attributed to metabolic disturbances caused by hepatic and/or renal failure, even if SAE generally precedes multi-organ failure.

Hypoperfusion consequent to septic shock can also cause brain damage and the effect of the use of sedative drugs should be also considered as possible cause of neurological impairment, since drugs often show delayed clearance in sepsis.

Anyway all the above mentioned conditions should be excluded before defining with the term of SAE a cerebral dysfunction which occurs in a septic patient.

## Clinical features

SAE generally occurs early during severe sepsis and precedes multiple-organ failure.

The most common clinical feature of SAE is the consciousness alteration which ranges from mildly reduced awareness to unresponsiveness and coma. Fluctuating confusional states, inattention and inappropriate behavior are often present in mild encephalopathic patients, mainly early in the course of the clinical syndrome. In more severe cases patients may present delirium, agitation and then consciousness deterioration and coma.

Some Authors use the term “delirium” instead of “encephalopathy” (14), but the two conditions are not synonymous since SAE is one of many causes of delirium, but delirium is not the only clinical presentation of SAE. Furthermore, symptoms of SAE can entirely bypass the stage of delirium (13).

Motor signs are uncommon; asterixis, myoclonus and tremors, which are frequent in metabolic encephalopathies, are rare in SAE. Relatively more common is the presence of a paratonic rigidity, defined as a velocity-dependent resistance to passive movements, less evident when limbs are moved slowly.

Seizures have been reported in SAE, but they are uncommon; cranial nerve dysfunction and lateralization are exceptional, and their presence require the exclusion of more frequent causes.

Last, neuroendocrine dysfunction and autonomic failure, which characterize severe sepsis are often a consequence of brain dysfunction (1–3).

GCS is used by many authors to describe cerebral impairment during sepsis, while the use of the Confusion Assessment Method for the Intensive Care Unit (CAM-ICU) is controversial (13–14).

## Diagnosis

### Instrumental findings

Diagnosis of SAE is primarily clinical and depends on the exclusion of other possible causes of brain deterioration (metabolic or structural).

EEG is almost sensitive and it can show abnormalities even when the neurological examination is normal (1). Young et al. identified 5 classes of progressively worsened EEG pattern related to worsened outcome: 1 = normal EEG, 2 = excessive theta, 3 = predominant delta, 4= triphasic waves, 5 = suppression or burst suppression, in ascending order of severity. Anyway it is not specific for SAE and it is often unavailable or unreliable for sedation or other metabolic derangement.

CT head scan generally is negative in case of SAE, but it should be performed to exclude a hypoxic/ischemic brain damage.

MRI can show brain abnormalities in case of SAE, but they are not specific for this condition.

In a case report previously published, we reported four cases of SAE (8); in two out of these MRI was negative, while in the other two patients it showed images of vasogenic edema (table 1). These images, like above mentioned, are more easily related to the loss of cerebral auto-regulation than to BBB disruption.

Bartinsky (15) reported 26 cases of PRES (posterior reversible encephalopathy syndrome) associated with isolate infection or sepsis. Fugate and colleagues identified 120 cases of PRES; in 7% of cases sepsis was the recognized cause. These patients had more often cortical involvement if compared to the other causes of PRES, and in all cases they had parieto-occipital involvement. Endothelial dysfunction was identified as the possible pathogenetic mechanism of PRES in septic patients (16). Sharshar et al. (17) described, in a MRI study of septic shock, injuries of the white matter in the “border” regions, where the terminal vessels may be easily jeopardized, independently of specific BBB lesions.

Anyway the cost of MRI and the complicated transport of the patients have been a limitation to its widespread use in the clinical management of sepsis.

Zauner et al. registered SEPs in septic patients and observed an increase of their peak latencies in cortical and subcortical pathways. This impairment was associated with severity of illness but the results did not differ significantly between patients with severe sepsis and those with septic shock (18).

Ohnesorge et al. registered SEPs in pigs with experimental systemic inflammatory response (SIRS): they observed an attenuation of amplitudes at least 4 hours before the hemodynamic SIRS criteria ensue. So SEPs can be considered like sensitive markers of developing cerebral dysfunction in SIRS (19).

Cerebrospinal fluid analysis does not give definitive information in case of SAE since it is generally normal, apart of an inconstant elevation of protein concentration. However it could be useful to exclude meningitis in suspected cases.

### Biomarkers

The markers of cerebral damage have been proposed for diagnosis of SAE. Among these S100B and NSE have been the most studied.

S100B is a calcium binding protein produced in the central nervous system mainly – but not exclusively – by astroglial cells. Its increase in serum and in CSF after brain injury has been attributed to an active secretion from these cells, since it probably takes part in the inflammatory response of the brain. It has been proposed as indicator of adverse neurocognitive outcome following global cerebral ischemia and brain trauma and as diagnostic marker of brain tumors and of BBB disruption.

NSE is the intracytoplasmatic glycolytic enzime enolase; it is found in neurons and neuroendocrine tissue and it is elevated in blood circulation after increase death rate of this cells.

Nguyen et al. (20) measured S100B and NSE in the serum of 170 patients with severe sepsis and septic shock. They found that both S100B and NSE increased and correlated with brain injury. S100B levels more closely reflected severe encephalopathy and type of brain lesions than NSE and GCS.

In contrast with these results, we previously reported data about serum S100B measurement in 19 patients with severe sepsis excluding those with septic shock (21); even if the protein was increased in 56% of patients, it didn’t correlate with the presence of SAE.

Since S100B has poor ability to escape the tight junctions of the blood brain barrier, its increase in the serum has been attributed to an increased permeability of the BBB. In porcine endotoxemic shock, Larsson et al. (22) found increased serum levels of S100B, which were attributed to minor BBB damage caused by the endotoxin effect on glial cell integrity, even if the Authors cannot exclude the contribution of other cells.

We observed in one patient normal CSF value of S100B with concomitant increased serum level; in this patient, serum origin of the protein is likely extracerebral and it cannot be attributed to alteration of BBB or direct brain damage (8).

## Prognosis

SAE is reversible even if survivors of severe sepsis have often long lasting or irreversible cognitive and behavioral sequel.

However the presence of SAE can have a negative influence on survival. This probably is due to the fact that the brain is not an isolated organ, but actively participates and controls the main functions of the body. The brain dysfunction which can occur during severe sepsis does not cause only consciousness alterations but, like previously reported, also dysfunctions in neuroendocrine and autonomic systems which can negatively influence the prognosis in septic patients.

## Management

At the moment a specific therapy of SAE does not exist; the outcome of patients depends on a prompt and appropriate treatment of sepsis as whole.

Some Authors obtained encouraging results from administration of branched-chain amino acids (13).

The blockade of C5a by administrating anti-C5a neutralizing antibodies, has been proposed, even if its real benefit is controversial (6, 23).

It has been demonstrated that Ghrelin prevents cognitive impairment in SAE by reducing neuronal apoptosis (24).

The use of Dotrecogin alpha has been suggested to reduce neuronal damage in human sepsis, even if its effect has been evaluated by measuring of serum S100B, which is not a reliable marker of SAE, like previously reported (25).

A more precise knowledge of pathophysiology of SAE will open new therapeutic perspective.

## Conclusions

Pathogenesis of SAE is multifactorial and it’s arduous to exclude an interplay between the toxic mediators involved in sepsis and the indirect effect of hypertermia, hypoperfusion and hypoxia.

Diagnosis of SAE is primary clinical, and there is no specific test. The role of S100B is not clear but its increase in serum is very probably of extracerebral origin and does not prove an injury of the BBB. Anyway the possibility that BBB damage is an important contributor of SAE deserves high regard. SAE is reversible, but it can negatively influence the prognosis in septic patients.

## Figures and Tables

**Table 1:** S100B serum and liquoral levels, EEG and MRI in 4 cases of sepsis

	Patient 1	Patient 2	Patient 3	Patient 4
Serum S100 B (μg/l)	0,72	0,12	0,46	0,2
Liquoral S100B (μg/l)	0,45	0,63	*	*
EEG (Young Classification)	3	1	3	3
MRI images	Negative	FLAIR and T2-hyperintense foci in the frontal regions	Negative	FLAIR and T2-hyperintense foci in periventricular regions

* not indicated
